# Closing immunization gap: Complete basic childhood vaccination and associated factors among children aged 12–23 months in Tanzania; a multilevel analysis of the 2022 Demographic and Health Survey

**DOI:** 10.1371/journal.pgph.0004810

**Published:** 2026-06-25

**Authors:** Erick Donard Oguma, Elihuruma Eliufoo Stephano, Sanun Ally Kessy, Jovin R. Tibenderana, Victoria Godfrey Majengo, Tegemea Patrick Mwalingo, Mussa Hassan Bago, Immaculata P. Kessy, Azan Abubakar Nyundo, Mtoro J. Mtoro

**Affiliations:** 1 Department of Clinical Nursing, School of Nursing and Public Health, The University of Dodoma, Iyumbu, Dodoma, Tanzania; 2 Directorate of Research and Training, Benjamin Mkapa Hospital, Iyumbu, Dodoma, Tanzania; 3 Department of Public Health, St, Francis University, College of Health and Allied Sciences, Ifakara, Tanzania; 4 Department of Obstetrics and Gynecology, Dodoma Regional Referral Hospital, Dodoma, Tanzania; 5 Department of Public Health and Community Nursing, School of Nursing and Public Health, The University of Dodoma, Iyumbu, Dodoma, Tanzania; 6 TILAM International, Department of Research and Surveillance, Dar es Salaam, Tanzania; 7 Department of Psychiatry and Mental Health, School of Medicine and Dentistry, The University of Dodoma, Iyumbu, Dodoma, Tanzania; PLOS: Public Library of Science, UNITED STATES OF AMERICA

## Abstract

Vaccination prevents an estimated 4–5 million deaths globally each year, including many among children under five. However, complete childhood vaccination coverage remains low in Sub-Saharan Africa, including Tanzania. This study examined complete basic vaccination coverage and associated factors among Tanzanian children aged 12–23 months. Analytical cross-sectional study of the 2022 Tanzania Demographic and Health Survey data was conducted. The sampling frame was stratified by geographic region and urban/rural areas, using a two-stage sampling method that selected primary sampling units based on census enumeration areas, followed by household selection using probability systematic sampling. Multilevel logistic regression, accounting for the complex survey design, was used to identify individual and community-level factors associated with complete childhood vaccination. Adjusted odds ratios (OR) and 95% Confidence intervals (CI) were used to estimate the strength of association. The prevalence of complete basic childhood vaccination was 52.5% (95%CI: 49.6 -55.3). Among these children, coverage for individual vaccines was high, with 86.5% receiving the first dose of measles and 91.0% receiving (Bacillus Calmette–Guérin) BCG. However, the percentage receiving the full dose was lower for polio (58.9%) and 90.0% for Diphtheria, Pertussis, and Tetanus (DPT). Mothers with primary, secondary/higher education, in middle wealth quintile, had more than four antenatal care (ANC) visits, had 1–2 under-five children had lower odds to completely vaccinate their children. At the community level, mothers in western, central zones and Zanzibar had higher odds to vaccinate their children. Complete basic childhood vaccination coverage in Tanzania was suboptimal and associated with various factors including maternal education, middle wealth, more ANC visits, and fewer young children exhibited higher odds of vaccination. Western, central zones, and Zanzibar showed higher coverage. Targeted interventions addressing education, wealth, ANC, family size, and regional disparities would be crucial to improve vaccination rates in Tanzania.

## Background

Immunization is one of the most cost-effective public health interventions, preventing an estimated 4.4 million deaths annually as of 2024 [1]. Despite ongoing efforts towards recovery and strengthening beyond pre-pandemic (2019) levels, a staggering 21 million children remained either unvaccinated or under-vaccinated in 2023 [1,2]. The number of children who did not receive any vaccines, often referred to as zero-dose children, reached 14.5 million in 2019 [3].

Incomplete childhood vaccination has been shown to raise the risk of acquiring and transmitting infections that may be prevented, which can cause severe illness, complications, and even death [4–6]. Furthermore, it may reduce herd immunity, which would facilitate the spread of infectious illnesses throughout the community [5,7].

Measles, polio, and diphtheria outbreaks are still a threat in many areas, and the World Health Organization (WHO) continues to emphasize the significance of attaining high immunization coverage to stop them. The WHO supports regional and national plans to accomplish this through frameworks such as the Measles and Rubella Strategic Framework and Immunization Agenda 2030 [8,9].

Despite the proven benefits of vaccination, coverage is still unequal, especially in developing countries where variations in uptake and access can pose serious public health issues [10,11]. In Africa, about one out of five children does not receive all of the necessary and basic vaccines. Consequently, each year, vaccine-preventable diseases (VPDs) continue to affect over 30 million children under five in Africa. Of these, over half a million of them die each year, which represents 58% of all VPD-related deaths worldwide [12]. The coverage of basic childhood vaccinations is low (59.4%) in Sub-Saharan Africa, with variation among the countries [13]. Complete basic childhood immunization is still a significant public health concern in East Africa, where it is low (69.21% in 2016), ranging from 39.5% in Ethiopia to 85% in Burundi [14].

In Tanzania, the routine childhood immunization schedule begins at birth with Bacillus Calmette–Guérin vaccination (BCG) and oral polio vaccine zero dose (OPV-0), followed by a primary series at 6, 10, and 14 weeks which includes OPV, rotavirus vaccine, pneumococcal conjugate vaccine (PCV) and pentavalent vaccine (DTP-HepB-Hib) which is a combination vaccine for (Diphtheria, Tetanus, Pertussis, Hepatitis B, and *Haemophilus influenzae* type b). A dose of inactivated polio vaccine (IPV) is also given during that same infancy schedule at 14 weeks. At 9 months, children receive the measles-rubella (MR) vaccine as part of the schedule. Additionally, a second dose of MR is scheduled at 18 months to further strengthen immunity [15]. Tanzania also introduced human papillomavirus (HPV) vaccination into its routine schedule for adolescent girls in 2018, often at the age of 14, using a two-dose schedule spaced six months apart [16].

According to Tanzania Demographic Health Survey (2022), 53% of children age 12–23 months are fully vaccinated against all basic antigens, which is a decline from 75% in 2015–16 (Received one dose each of BCG and measles vaccines, and three doses each of polio and pentavalent (Diphtheria, Tetanus, Pertussis, *Haemophilus influenzae* type b, and Hepatitis B) [17]. However, 23% of children aged 12–23 months are fully vaccinated according to the national schedule. Across regions, vaccination coverage ranges from 3% in Shinyanga to 66% in Kilimanjaro [17].

To improve immunization rates and decrease health disparities, public health policies must take into account the current state of basic childhood vaccination coverage and its associated factors. Therefore, this multilevel analysis aims to evaluate the coverage of complete basic vaccination and its associated factors among children aged 12–23 months in Tanzania using the 2022 Demographic and Health Survey. The study seeks to provide valuable insights for policymakers and health practitioners that will help to strengthen immunization efforts in Tanzania.

## Methods

### Study setting

Tanzania is a country located in East Africa with a population of approximately 61.7 million people [18]. It is made up of 26 regions, which are divided into both mainland Tanzania and Zanzibar, an archipelago consisting of two main islands, Unguja and Pemba. Zanzibar has a semi-autonomous government and its own health system, which operates slightly differently from mainland Tanzania.

Regarding vaccination, Tanzania follows the World Health Organization’s Expanded Program on Immunization (EPI), with vaccines such as BCG, polio, DTP, Hib, hepatitis B, and measles included in the national schedule [19]. Zanzibar also adheres to this schedule, but due to its autonomous health system, there may be some differences in the logistics and implementation of these programs compared to mainland regions.

### Data source and design

An analytical cross-sectional study was conducted using the secondary data from the 2022 TDHS. The TDHS is nationally representative household survey that collect demographic information and various health indicators. The survey was conducted by the Tanzania National Bureau of Statistics, collaborating with the Ministries of Tanzania Mainland and Zanzibar.

### Population and sampling

Data for this study were obtained from the recent DHS conducted between 24 February and 21 July 2022 across all regions in Tanzania. The overall survey response rate was 97%. The target population for the 2022 TDHS included women of reproductive age (15–49 years), children, men and household across all regions in Tanzania. For the purposes of this analysis, the focus was specifically on mothers with children aged 12–23 months.

The 2022 TDHS employed a two-stage stratified complex sampling design. In the first stage, the country was stratified into urban and rural areas. In the second stage, primary sampling units (PSUs) were selected from each stratum, followed by the selection of households within those PSUs. All women who spent the night in the selected households prior to the survey were eligible for the survey. Our analysis utilized the Kids Record file (KR), which initially contained 10,783 records. We excluded children who had died, those under 12 months of age, and those aged ≥24 months, resulting in a final weighted sample of 2,180 children.

### Study variables

#### Outcome variable.

The dependent variable was complete basic childhood vaccination status among children aged 12–23 months. According to DHS Report, complete basic childhood vaccination refers to a child receiving one dose of BCG vaccine, three doses of pentavalent vaccines (diphtheria, tetanus, pertussis, *Haemophilus influenzae* type b, and hepatitis B), three doses of polio vaccine, and one dose of measles vaccine before the age of 12 months. Children who received all these recommended doses were categorized as “yes” (fully vaccinated), while those who did not were categorized as “no” (not fully vaccinated). Information on child vaccination was obtained from mothers’ verbal reports and data extracted from childhood immunization cards. In this study, 81% of children aged 12–23 months had vaccination cards available at the time of the survey.

#### Explanatory variables.

Explanatory variables were categorized into individual and community levels based on available data and relevant literature.

**Individual level-characteristics:** Individual level characteristics included both maternal and child characteristics.

Maternal characteristics; age in years (15–24, 25–34 or 35–49), marital status (never married, married/cohabiting and previously married), education level (no formal education, primary education and secondary/higher), literacy (literate or illiterate), wealth index (poorest, poorer, middle, richer and richest), media exposure ((yes as listening to the radio, reading the newspaper or watching Television less than once a week or at least once a week or no if otherwise), working status (working or not working), sex of household head (male or female), household members (<6 or ≥6), ANC visits (<4 or ≥4), place of delivery (home or health facility), parity (primiparous, multiparous and grand multiparous), Post-natal care (PNC) checkup (yes or now), distance to the facility (big problem or nor a big problem).

Children’s related characteristics; child sex (male or female), birth order (1st, 2nd & 3rd, or 4th & more), and number of additional under-five children in the household (none, 1–2, and ≥3).

**Community-level characteristics:** Community level characteristics; place of residence (urban or rural), geographical zones (western, northern, central, southern highlands, southern, southwest highlands, lake, eastern and Zanzibar). Community literacy was calculated based on the proportion of women in each cluster based on literacy category, classified as either low (communities where <50% of women are literate) or high (communities where ≥50% of women are literate).

### Data management and analysis

To account for the complex survey design, we applied individual sampling weights (v005/1,000,000), primary sampling units (clusters; v021), and stratification variable (v023) to adjust for clustering. These adjustments help minimize sampling bias and ensure that the estimates are nationally representative. Initial weights in a TDHS dataset are pre-computed from the sample design and response rates, The DHS Program then normalizes these weights by dividing by their average, making the sum of the normalized weights equal the total number of cases.

The survey design was declared in Stata using the *svyset* command, and descriptive analyses were conducted with the svy prefix. Statistical analyses were performed in Stata version 18.5 (STATA Corp, College Station, TX).

Descriptive statistics were summarized using means, standard deviation (SD), medians, frequency, and proportion for categorical variables. The Pearson chi-square test was used to compare the differences in the proportion of vaccination status across participants’ characteristics. Given the hierarchical structure of the DHS data, where women are nested within households, and households within clusters, individual within the same cluster tend to be more similar to each other. This clustering violates the assumption of independent observations and homogeneity of variance across clusters. Therefore, we used weighted multilevel mixed-effects logistic regression. Four models were applied: the null model (outcome variable only), Model I (only individual-level factors), Model II (only community-level factors), and Model III (both individual and community-level factors. The *melogit* package in Stata was used in fitting these models.

Random effects were used to estimate the mean distribution of effects and compare individuals from two randomly chosen clusters. Intra-class correlation coefficient (ICC), Median odds ratio (MOR), and Proportional Change in Variance [20–22]. The model that best suited the data was the one with the lowest deviance (Model III). Adjusted Odds ratio (AOR) and corresponding 95% confidence intervals (CI) were presented to estimate the magnitude and strength of the association. Model selection was based on the Akaike Information Criterion (AIC). Given that odds ratios from logistic regression may overestimate associations when the outcome is not rare (>10%), we compared the modified Poisson regression and logistic regression models. The logistic regression model had the lowest AIC, indicating superior model fit, and was therefore selected. A variance inflation factor (VIF) was used to assess for multicollinearity between independent variables before fitting a multivariable regression model. The mean VIF was < 10 indicating no significant multicollinearity. Missing values were minimal (<2% for most variables). Consequently, complete case analysis was applied in the regression modelling, as no indication of systematic bias was observed in the missing data. All tests were two-tailed and statistical significance was considered for a p-value < 0.05.

### Ethics approval and consent to participate

This study utilized publicly available, de-identified data from the 2022 TDHS, accessible online through the DHS program. The original survey received ethical approval from both the National Institute of Medical Research Ethics Committee in Tanzania and the ICF Macro Ethics Committee in Calverton, New York. Permission to use the data for this secondary analysis was granted by the DHS program upon acceptance of the proposed analysis plan under the designated account, with credentials available upon request via https://dhsprogram.com/data/dataset_admin/index.cfm. As this study involved secondary data analysis of publicly accessible datasets, no additional ethical approval was required. Informed consent was obtained from all participants during the initial survey, and all procedures adhered strictly to relevant guidelines and regulations. Further details regarding DHS data usage and ethical standards can be found at http://goo.gl/ny8T6X.

## Results

### Sociodemographic characteristics of study participants

The mean age of mothers who had their child vaccinated was 28.7 years (standard deviation = 6.9), with 44.0% aged 25–34 years. The majority (83.6%) were married or cohabiting, and just 7.0% were never married. More than half (54.9%) had completed primary education, and 71.6% were literate. Regarding socioeconomic status, 23.6% were from the poorest and 18.9% from the richest households. Nearly two-thirds (60.5%) were working, and 62.9% were exposed to media. Most (81.6%) were delivered at the health facility, and 62.3% attended more than four ANC visits. More than half of the children were male, and 21.9% were firstborn. More than half (72.4%) were from a rural setting, and 33.8% from the lake zone. There was a significant difference in individual and community level characteristics (p < 0.05) with education, literacy, wealth index, media exposure, ANC visits, place of delivery, and number of under-five children. (Table 1).

**Table 1:** Individual and community level characteristics and distribution of vaccination status among children aged 12-23 months in Tanzania (N = 2,180).

Characteristics	N (%)	Vaccination status, N (%)		p-value
		**Partial**	**Complete**	
**Mother’s age (years)**				0.769
15-24	732 (33.6)	355 (48.5)	377 (51.5)	
25-34	960 (44.0)	457 (47.6)	503 (52.4)	
35-49	488 (22.4)	224 (45.9)	264 (54.1)	
Mean (±SD)				
**Marital Status**				0.137
Never married	154 (7.0)	62 (40.0)	92 (60.0)	
Married/Cohabiting	1822 (83.6)	868 (47.6)	954 (52.4)	
Previously married	205 (9.4)	107 (52.1)	98 (47.9)	
**Education Level**				<0.001
No formal education	483 (22.2)	275 (56.9)	208 (43.1)	
Primary	1197 (54.9)	562 (46.9)	635 (53.1)	
Secondary/Higher	501 (23.0)	200 (40.0)	301 (60.0)	
**Literacy**				0.001
Illiterate	620 (28.4)	344 (55.5)	276 (44.5)	
Literate	1561 (71.6)	693 (44.3)	868 (55.7)	
**Wealth index**				0.004
Poorest	514 (23.6)	296 (57.5)	218 (42.5)	
Poorer	438 (20.1)	196 (44.8)	242 (55.2)	
Middle	392 (18.0)	170 (43.3)	222 (56.7)	
Richer	424 (19.4)	187 (43.9)	237 (56.1)	
Richest	413 (18.9)	188 (45.6)	225 (54.4)	
**Media Exposure**				0.008
No	810 (37.1)	420 (51.8)	390 (48.2)	
Yes	1371 (62.9)	615 (45.0)	754 (55.0)	
**Working Status**				0.601
Not working	861 (39.5)	418 (48.5)	443 (51.5)	
Working	1319 (60.5)	618 (46.9)	701 (53.1)	
**Sex of Household Head**				0.721
Male	1744 (80)	833 (47.7)	911 (52.3)	
Female	437 (20)	203 (46.6)	233 (53.4)	
**Household members**				0.175
< 6	1021 (46.8)	465 (45.5)	556 (54.5)	
≥ 6	1159 (53.2)	572 (49.3)	588 (50.7)	
**Antenatal Care (ANC) Visits**				0.012
< 4	821 (37.7)	423 (51.5)	398 (48.5)	
≥ 4	1359 (62.3)	613 (45.1)	746 (54.9)	
**Place of delivery**				0.012
Home	401 (18.4)	222 (55.3)	179 (44.7)	
Health facility	1779 (81.6)	814 (45.7)	965 (54.3)	
**Parity**				
Primiparous	445 (20.4)	205 (46.1)	240 (53.9)	0.854
Multiparous	1162 (53.3)	554 (47.7)	608 (52.3)	
Grand multiparous	574 (26.3)	278 (48.3)	296 (51.7)	
**Postnatal care (PNC) check-up (n = 1,675)**				0.356
No	635 (37.9)	278 (43.8)	357 (56.2)	
Yes	1040 (62.1)	488 (46.9)	552 (53.1)	
**Child sex**				0.451
Male	1139 (52.3)	530 (46.5)	610 (53.5)	
Female	1041 (47.7)	507 (48.6)	534 (51.4)	
**Birth order**				0.896
1st	477 (21.9)	224 (46.7)	253 (53.3)	
2nd & 3rd	873 (40.0)	412 (47.2)	461 (52.8)	
4th & more	831 (38.1)	401 (48.3)	430 (51.7)	
**Number of additional under-five children**				0.041
None	48 (2.2)	27 (57.6)	21 (42.4)	
1-2	1,736 (79.6)	796 (45.9)	940 (54.1)	
≥ 3	397 (18.2)	214 (53.5)	183 (46.5)	
**Distance to the facility**				0.107
Big problem	734 (33.7)	373 (50.8)	361 (49.2)	
Not a big problem	1,446 (66.3)	663 (45.8)	783 (54.2)	
**Residence**				0.593
Urban	603 (27.6)	279 (46.2)	324 (53.8)	
Rural	1,578 (72.4)	757 (48.0)	820 (52.0)	
**Geographical Zones**				0.055
Western	230 (10.5)	109 (47.6)	121 (52.4)	
Northern	226 (10.4)	105 (46.4)	121 (53.6)	
Central	222 (10.2)	92 (41.6)	130 (58.4)	
Southern highlands	124 (5.7)	45 (36.6)	79 (63.4)	
Southern	84 (3.9)	50 (59.4)	34 (40.6)	
Southwest highlands	200 (9.2)	108 (53.7)	92 (46.3)	
Lake	737 (33.8)	362 (49.0)	375 (51.0)	
Eastern	298 (13.7)	148 (46.0)	150 (50.4)	
Zanzibar	61 (2.8)	19 (30.3)	42 (69.7)	

### Prevalence of complete basic vaccination

**Fig 1:**
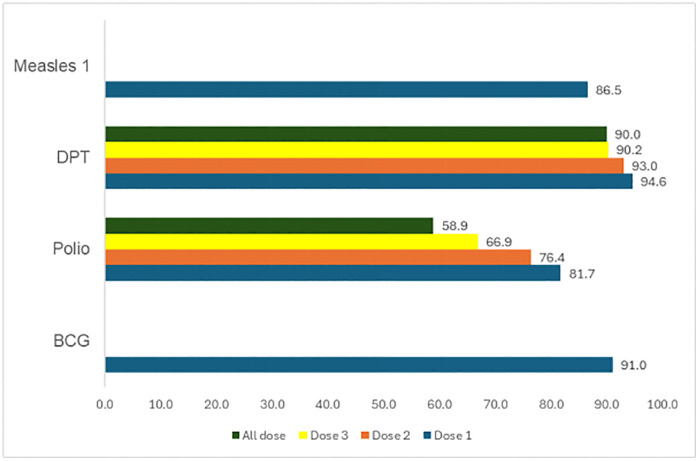
Distribution of basic vaccination among children aged 12-23 months in Tanzania.

The overall prevalence of complete basic vaccination among children aged 12–23 months was 52.5% (95% CI: 49.6-55.3). Among these children, coverage for individual vaccines was high, with 86.5% receiving the first dose of measles and 91.0% receiving BCG. However, the percentage receiving the full series was lower for polio (58.9% for all three doses) and DPT (90.0% for all three doses) (Fig 1).

### Factors associated with vaccination status

From the final multivariable analyses, mothers with primary education (AOR = 1.51, 95%CI: 1.16-1.99) and secondary/higher (AOR = 1.92, 95%CI: 1.35-2.74) had higher odds to vaccinate their children than their counterparts. Mothers with more than four ANC visits had 1.25 times higher odds to attain complete vaccination than their counterparts (AOR = 1.25, 95%CI:1.02-1.53). Households with 1–2 under-five children had 2.23 times higher odds of vaccinating their children compared to those with no under-five children (AOR = 2.23, 95%CI: 1.19-4.15). Geographically, mothers in the western zone (AOR = 2.18, 95%CI: 1.13-4.22), central zone (AOR = 2.13, 95%CI: 1.11-4.07), and Zanzibar (AOR = 3.12, 95%CI: 1.64-5.92) had higher odds to vaccinate their children than those in the southern zone. (Table 2).

**Table 2:** Individual and community factors associated with complete vaccination among children aged 12-23 months in Tanzania.

Characteristics	Model 0	Model I	Model II	Model III
		**AOR (95%CI)**	**AOR (95%CI)**	**AOR (95%CI)**
**Individual-level**				
**Mother’s age (years)**				
15-24		Ref		Ref
25-34		1.01 (0.76–1.31)		1.02 (0.78–1.34)
35-49		1.15 (0.79–1.66)		1.20 (0.83–1.74)
**Education Level**				
No formal education		Ref		Ref
Primary		1.57 (1.21–2.04)*		1.51 (1.16–1.99)*
Secondary/Higher		2.34 (1.67–3.27)*		1.92 (1.35–2.74)*
**Wealth index**				
Poorest		Ref		Ref
Poorer		1.29 (0.96–1.75)		1.26 (0.93–1.71)
Middle		1.38 (1.01–1.89)*		1.32 (0.76–0.95)*
Richer		1.31 (0.94–1.84)		1.27 (0.88–1.83)
Richest		1.08 (0.75–1.56)		1.04 (0.68–1.60)
**ANC Visits**				
< 4		Ref		Ref
≥ 4		1.26 (1.03–1.55)*		1.25 (1.02–1.53)*
**Place of delivery**				
Home		Ref		Ref
Health facility		1.06 (0.81–1.38)		1.10 (0.84–1.45)
**Child sex**				
Male		Ref		Ref
Female		0.99 (0.83–1.21)		0.99 (0.82–1.20)
**Birth order**				
1st		Ref		Ref
2nd & 3rd		0.93 (0.70–1.23)		0.90 (0.68–1.19)
4th & more		1.10 (0.76–1.59)		0.99 (0.68–1.43)
**Number of additional under-five children**				
None		Ref		Ref
1-2		2.15 (1.16–3.99)*		2.23 (1.19–4.15)*
≥ 3		1.52 (0.79–2.91)		1.55 (0.80–2.97)
**Community literacy**				
Low			Ref	Ref
High			1.52 (1.15–2.00)*	1.17 (0.87–1.56)
**Residence**				
Urban			Ref	Ref
Rural			0.97 (0.75–1.25)	1.07 (0.79–1.46)
**Geographical Zones**				
Western			1.74 (0.90–3.35)	2.18 (1.13–4.22)*
Northern			1.82 (0.95–3.50)	1.92 (0.99–3.70)
Central			1.96 (1.03–3.74)*	2.13 (1.11–4.07)*
Southern highlands			2.19 (1.13–4.26)*	2.23 (1.15–4.34)
Southern			Ref	Ref
Southwest highlands			1.27 (0.68–2.34)	1.32 (0.71–2.45)
Lake			1.37 (0.78–2.45)	1.60 (0.89–2.87)
Eastern			1.21 (0.64–2.27)	1.28 (0.68–2.42)
Zanzibar			3.24 (1.76–5.98)*	3.12 (1.64–5.92)*
**Random effects**				
Variance	0.65	0.50	0.48	0.43
PCV	Ref	23.1%	21.2%	33.8%
ICC	16.5%	13.3%	12.7%	11.6%
MOR	1.31	1.28	1.27	1.26
**Model fitness**				
AIC	2900.32	2866.53	2870.98	2855.12
BIC	2911.66	2962.92	2939.02	3008.21
Deviance	2896.32	2832.53	2846.98	2801.12

Key:*p < 0.05, ICC: Intra-class Correlation Coefficient, PCV: Proportional Change in Variance, MOR: Median Odds Ratio, AIC: Akaike Information Criterion, BIC: Bayesian Information Criterion, Ref: Reference category.

### Random effects and model fitness

The null model showed a variance of 0.65 and a p-value of < 0.001, indicating significant differences in complete vaccination across localities. In this model, the odds of complete vaccination varied by a factor of 1.31 (MOR). According to the Intraclass Correlation Coefficient (ICC) in Model I, 13.3% of the variation in complete vaccination was attributed to individual differences. In Model I, the odds of complete vaccination was 1.28 times higher than for incomplete vaccination. The best-fitting model was Model III, which had the lowest deviance (2801.12) and AIC (2855.12). (Table 2).

## Discussion

Childhood immunization remains a critical intervention for preventing and protecting children from preventable childhood infections [16]. However, incomplete childhood vaccination remains a global concern in developing countries [6,12,23]. This study intends to evaluate the coverage of complete basic vaccination and its associated factors among children aged 12–23 months in Tanzania. The study seeks to generate evidence-based recommendations that will aid in developing cost-effective public interventions and inform health policy.

The study revealed that more than fifty percent of children, 52.5%, had completed their basic vaccination. Full basic vaccination coverage among 12–23 months children in this study reveals a substantial decline of 22.5% from 75% in the previous Survey 2015–16 [17]. The possible reason for this sudden drop may be influenced by COVID-19 disruption, an increase in population, geographical access barriers, and rural-urban disparities.

Furthermore, inconsistency in complete basic vaccination coverage was observed in previous studies conducted in Ethiopia, Malawi, and East Africa, these studies reported high prevalence of complete basic childhood vaccination, above 69% [14,24,25]. The discrepancy in these findings might be attributed by their variation in sample size and study setting. Low prevalence of complete basic vaccination in this study creates the need for immediate public intervention. As evidenced, incomplete childhood vaccination has been shown to raise the risk of acquiring infections that may be prevented, which can cause severe sickness, complications, and even death [4–6]. Furthermore, incomplete vaccination schedule may reduce herd immunity, which would facilitate the spread of infectious illnesses throughout the community [5,7].

Moreover, the finding revealed that mothers with primary and secondary or higher had higher odds to vaccinate their children completely than women with no education. These findings are consistent with those reported from the study done in Mozambique, Ethiopia, East Africa, and Ghana, which also reported high education to be associated with full vaccination among children [14,26–28].

The possible reason could be that mothers who were less educated or never attended school had higher odds to be less informed or aware of the health benefits of childhood immunization and the negative consequences of incomplete childhood immunization. Additionally, low education has been reported to be associated with negative perception toward childhood immunization, poor child clinic attendance, and little child health attention, which further increases the chance of incomplete childhood vaccination [23,29].

Additionally, the systematic review highlighted a lack of information or knowledge as a significant factor influencing parents’ views and practices around routine childhood vaccination [30]. This knowledge gap often leads to hesitation or refusal of vaccinations, particularly in communities where educational outreach and health communication are limited.

Based on these results, the study underscores the importance of providing community education on childhood immunization while putting more effort into educating women on the benefits of full vaccination and the effects of incomplete childhood vaccination. This would be beneficial if women were informed early; therefore, effective integration of immunization education in antenatal care would be a good starting point.

Moreover, the study revealed that mothers who have attended four antenatal care visits and above had higher odds to complete vaccinating their children than those who attend fewer than four visits. These results align with the findings reported from the previous study in Ethiopia, East Africa, and Ghana [14,31]. The similarity in these studies could be explained by the fact that women who attend more than 4 ANC visits had higher odds to receive health information (education) about their expected future baby, including immunization and breastfeeding. Hence, emphasizing that women to attend antenatal care is one of the key aspects in maximizing full vaccination coverage.

Similarly, the study revealed the wealth index as one of the important determinants of complete basic vaccination in children. The finding shows that mothers with a middle wealth index had higher odds to fully vaccinate their children compared to mothers with a poor wealth index. This finding is supported by the study conducted in Malawi and East Africa [14,25].

Due to the cost incurred during transport, Poor economic status may limit a mother from accessing the health Centre, particularly women who live a far distance from the health facility [24,26,28]. This may expose these women to a high risk of missing some vaccine. The situation will even be more severe for single-mother families and unmarried mothers who are the primary providers in their family [24,32]. Because of financial insecurity, they are spending most of their time looking for money to take care of their family, which may minimize their attention on child health matters, such as taking a child to clinics. They may delegate this responsibility to the house girls most of the time.

This study underscores the importance of empowering women on major economic issues. This could be achieved by creating equal opportunity, minimizing barriers to access to economic activities, providing affordable capital for economic development, providing more economic activities, and strengthening gender mainstreaming.

Furthermore, the study revealed that households with 1–2 under-five children had higher odds to fully vaccinate their children than those without under-five children. This finding correlates with the previous studies conducted in Somalia [33]. The possible reason for this similarity is that women with 1–2 children have higher odds to have vaccination experience than those without children or who had never been exposed to vaccination issues. Prior childhood immunization experience may influence their decision whether their child should be fully vaccinated. Additionally, these mothers may have more knowledge about childhood vaccination, which may affect their decision.

Additionally, the study shows that although there is a high initial vaccination uptake (e.g., 91% for BCG, 86.5% for measles), only 52.5% of children obtain all of the recommended basic immunizations, with notable declines for vaccines such as polio (58.9%). This emphasizes the need for policies that increase health education, especially for mothers with less education and fewer ANC visits, strengthen follow-up procedures, and address the challenges presented by lower-income and larger households. Regional differences also highlight the significance of duplicating effective models from high-coverage zones, such as the central region and Zanzibar, in order to adapt methods to underperforming areas. Boosting full vaccination coverage will require improving community participation, boosting access, and integrating immunization with mother and child health services.

### Strengths and limitations

This study’s strengths include using nationally representative 2022 TDHS data and a large sample, enhancing the generalizability of our findings to the Tanzanian population. The rigorous data collection, involving experienced field assistants, ensured high data quality and reliability. Moreover, multilevel binary logistic regression provided a robust analysis, effectively accounting for survey design complexities and strengthening the validity of our conclusions.

However, this study is the potential for recall bias, especially when vaccination cards were not available for review. This may lead to inaccuracies in reporting vaccination histories, as caregivers may struggle to accurately recall immunization events. Additionally, the small sample size at the regional and provincial levels may limit the generalizability of the findings and reduce the statistical power to detect regional variations in vaccination coverage. These factors should be considered when interpreting the results, as they may affect the reliability of the data at finer geographical levels.

Another limitation relates to the high prevalence of the outcome (52.5%), which may cause adjusted odds ratios to overstate the magnitude of associations. Because odds ratios do not directly translate into changes in risk or probability when outcomes are common, they should be interpreted with caution. Accordingly, we report findings in terms of higher or lower odds rather than precise risk estimates. The model fit statistics (AIC) indicated that the logistic regression model provided the best fit for this data, supporting the robustness of the analytical approach.

### Implications for practice and policy recommendations

The suboptimal rate of complete basic childhood vaccination in Tanzania necessitates targeted and multi-faceted interventions. Given the positive influence of maternal education, healthcare providers should prioritize clear and accessible vaccination information during antenatal and postnatal care visits, tailoring messages to mothers with varying levels of education. Furthermore, the higher coverage observed in the western, central zones, and Zanzibar warrants investigation to identify and replicate successful local practices in other regions. To improve complete childhood vaccination coverage, policymakers should invest in and strengthen initiatives promoting girls’ education and women’s literacy nationwide. Targeted financial mechanisms would be essential to ensure equitable access to vaccination services for vulnerable populations. Integrating comprehensive immunization education into enhanced antenatal care services is essential.

## Conclusion

Complete basic childhood vaccination coverage in Tanzania was suboptimal and associated with various factors, including maternal education, middle wealth, more ANC visits, and fewer young children exhibiting higher odds of vaccination. Western, central zones, and Zanzibar showed higher coverage. Targeted interventions addressing education, wealth, ANC, family size, and regional disparities would be crucial to improving vaccination rates in Tanzania.

## Supporting information

S1 Data Data template of study participants. (SAV)

## Abbreviations

AIC: Akaike Information Criterion
ANC: Antenatal Care
AOR: Adjusted odds ratio
BCG: Bacillus Calmette-Guérin
BIC: Bayesian Information Criterion
DHS: Demographics and Health Survey
DPT: Diphtheria-Pertussis-Tetanus
EAs: Enumeration areas
ICC: Intra-class Correlation Coefficient
LLR: Likelihood ratio
MOR: Median Odds Ratio
PCV: Proportional Change in Variance
PNC: Postnatal care
PSU: Primary sampling unit
SSA: Sub-Saharan Africa
TDHS: Tanzania Demographics and Health Survey
VIF: Variance inflation factor
